# Neuroanatomical correlates of speech and singing production in chronic post-stroke aphasia

**DOI:** 10.1093/braincomms/fcac001

**Published:** 2022-01-11

**Authors:** Noelia Martínez-Molina, Sini-Tuuli Siponkoski, Anni Pitkäniemi, Nella Moisseinen, Linda Kuusela, Johanna Pekkola, Sari Laitinen, Essi-Reetta Särkämö, Susanna Melkas, Boris Kleber, Gottfried Schlaug, Aleksi Sihvonen, Teppo Särkämö

**Affiliations:** 1 Music, Ageing and Rehabilitation Team, Cognitive Brain Research Unit, Department of Psychology and Logopedics, University of Helsinki, Helsinki, Finland; 2 Department of Physics, University of Helsinki, Helsinki, Finland; 3 HUS Medical Imaging Center, Department of Radiology, Helsinki Central University Hospital and University of Helsinki, Helsinki, Finland; 4 Espoo Hospital, Espoo, Finland; 5 Private Choir Conductor, Vantaa, Finland; 6 Department of Neurology, University of Helsinki and Helsinki University Hospital, Helsinki, Finland; 7 Center for Music in the Brain, Department of Clinical Medicine, Aarhus University and The Royal Academy of Music, Aarhus/Aalborg, Denmark; 8 Department of Neurology, UMass Medical School—Baystate and Institute of Applied Life Sciences, UMass Amherst, Amherst, MA, USA; 9 Centre for Clinical Research, The University of Queensland, Brisbane, Australia

**Keywords:** aphasia, lesion-symptom mapping, singing, speech, voxel-based morphometry

## Abstract

A classical observation in neurology is that aphasic stroke patients with impairments in speech production can nonetheless sing the same utterances. This preserved ability suggests a distinctive neural architecture for singing that could contribute to speech recovery. However, to date, these structural correlates remain unknown. Here, we combined a multivariate lesion–symptom mapping and voxel-based morphometry approach to analyse the relationship between lesion patterns and grey matter volume and production rate in speech and singing tasks. Lesion patterns for spontaneous speech and cued repetition extended into frontal, temporal and parietal areas typically reported within the speech production network. Impairment in spontaneous singing was associated with damage to the left anterior–posterior superior and middle temporal gyri. Preservation of grey matter volume in the same regions where damage led to poor speech and singing production supported better performance in these tasks. When dividing the patients into fluent and dysfluent singers based on the singing performance from demographically matched controls, we found that the preservation of the left middle temporal gyrus was related to better spontaneous singing. These findings provide insights into the structural correlates of singing in chronic aphasia and may serve as biomarkers to predict treatment response in clinical trials using singing-based interventions for speech rehabilitation.

## Teaser

Martínez-Molina *et al*. used a combination of support vector regression-based multivariate lesion-symptom mapping and voxel-based morphometry in patients with chronic post-stroke aphasia. Both analytical approaches showed that while spontaneous speech and cued repetition are associated with left frontotemporal and parietal regions, spontaneous singing is associated with left temporal regions.

## Graphical Abstract

**Graphical Abstract:**
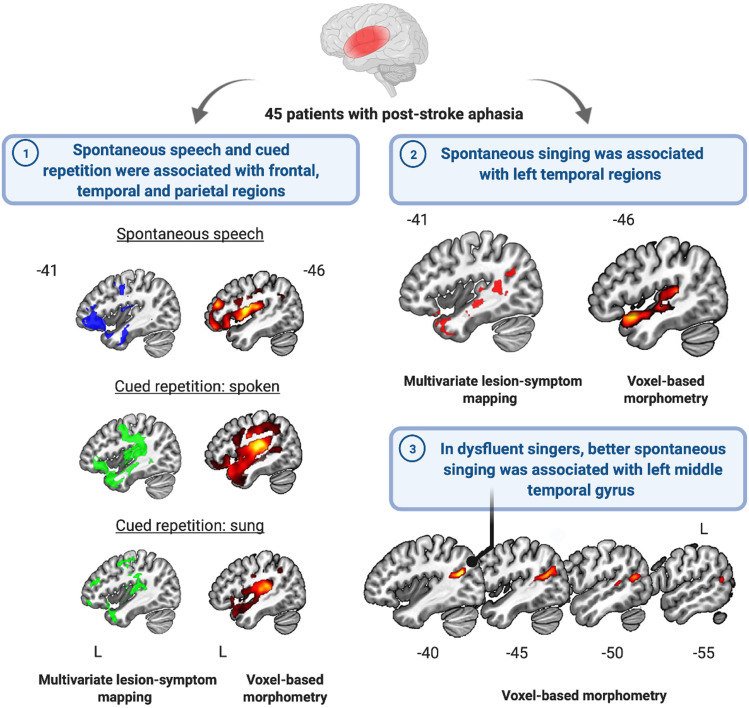

## Introduction

Amongst stroke survivors, 35% experience aphasia, a debilitating loss of linguistic processing and production abilities, even 1 year after the stroke and speech therapy.^1^ Despite impairments in speech production, a classical observation in neurology has been the relative preservation of singing ability in aphasic patients.^2^ Although the neural basis for singing in chronic post-stroke aphasia remains elusive, existing evidence suggests that singing could serve as a therapeutic tool to promote speech recovery. In non-fluent aphasic patients, some studies have highlighted the potential of singing for improving the production of verbal material, especially when produced along an auditory model.^3^ Recent evidence also suggests that sung presentation of novel short stories enhances their learning and recall in mild chronic aphasia.^4,5^ Similarly, melodic intonation therapy (MIT), which involves melodic intoning of formulaic phrases coupled with rhythmic left-hand tapping, has been reported to improve speech production in chronic aphasia.^6^

During the past 15 years, structural and functional neuroimaging studies have begun to map the neural basis of singing perception and production in the healthy brain, especially regarding its laterality and relationship with speech. Across functional imaging studies, a common finding has been that the neural processing of singing and speaking shows an extensive overlap, both engaging largely bilateral frontal, temporal and parietal brain regions and sharing a common vocal production network.^7–10^ Similarly, studies of singing-related neuroplasticity in healthy subjects have reported structural changes in left,^11^ right^12^ or bilateral^13^ frontotemporal regions or white matter pathways, such as the arcuate fasciculus. In stroke patients, our recent functional MRI (fMRI) study, which used a passive listening task, provided novel evidence that sung music activates more extensive bilateral frontotemporal regions than instrumental music at the early post-stroke stage, also in aphasic patients.^14^ Structural and functional changes in both left and right hemisphere have also been linked to the efficacy of music-based aphasia rehabilitation utilizing singing^15–20^ or vocal music listening.^21,22^

In aphasic patients, structural and fMRI could be used to explore the neural basis of singing ability, but this has not been done before. Compared with task-based fMRI, which is challenging in aphasic patients, structural MRI is more routinely acquired and, when coupled with behavioural data, it enables voxel-based lesion-symptom mapping (VLSM) and voxel-based morphometry (VBM) analyses that can reveal brain areas linked to behavioural performance. While VLSM and VBM approaches have been successfully used to map the lesioned and intact brain regions crucial for speech production,^23,24^ virtually nothing is known about the neuroanatomical substrates of singing production in aphasia. Given this scarce evidence, it would be crucial to apply VLSM and VBM analyses through which to map the structural mechanisms for impaired/preserved singing ability in aphasia and to identify putative biomarkers for predicting outcomes in longitudinal singing-based interventions.

Here, we adopted a dual approach to evaluate the critical lesion patterns and intact brain regions associated with both free (spontaneous) and cued (repetition) production of speech and singing in aphasia using production rate, which is a commonly used measure of fluency in language impairments.^25^ Using a sample of stroke patients with chronic aphasia (*N* = 45), we (i) applied a support vector regression-based multivariate lesion–symptom mapping technique (SVR-LSM) to define the lesion locations associated with poor speech or singing production and (ii) used VBM to explore regional grey matter volumes (GMVs) associated with performance in these tasks. We also performed a stepwise regression analysis including clinical, demographic, musical information as well as lesion load on 23 grey matter (GM) regions within the vocal motor production network to investigate the most important predictors for spontaneous singing in post-stroke aphasia. Finally, we divided the aphasic patients into fluent and dysfluent singers based on how well they were able to produce words when singing compared with healthy control subjects to test for potential neuroanatomical differences in GMV related to singing production. We hypothesized that damage to areas in motor, somatosensory and temporal regions, which are structurally malleable by singing experience,^12^ would be particularly detrimental to achieve normal singing fluency. We also anticipated that GMV in the same areas in either hemisphere would play an important role in singing production as it has been suggested that speaking and singing could be sharing a bilateral network for vocal production.^7–10^ Finally, we explored whether dividing our patients into fluent and dysfluent singers based on normative data from a demographically matched sample of healthy participants would provide additional information regarding the neuroanatomical correlates of singing fluency.

## Materials and methods

### Participants

**Figure 1:**
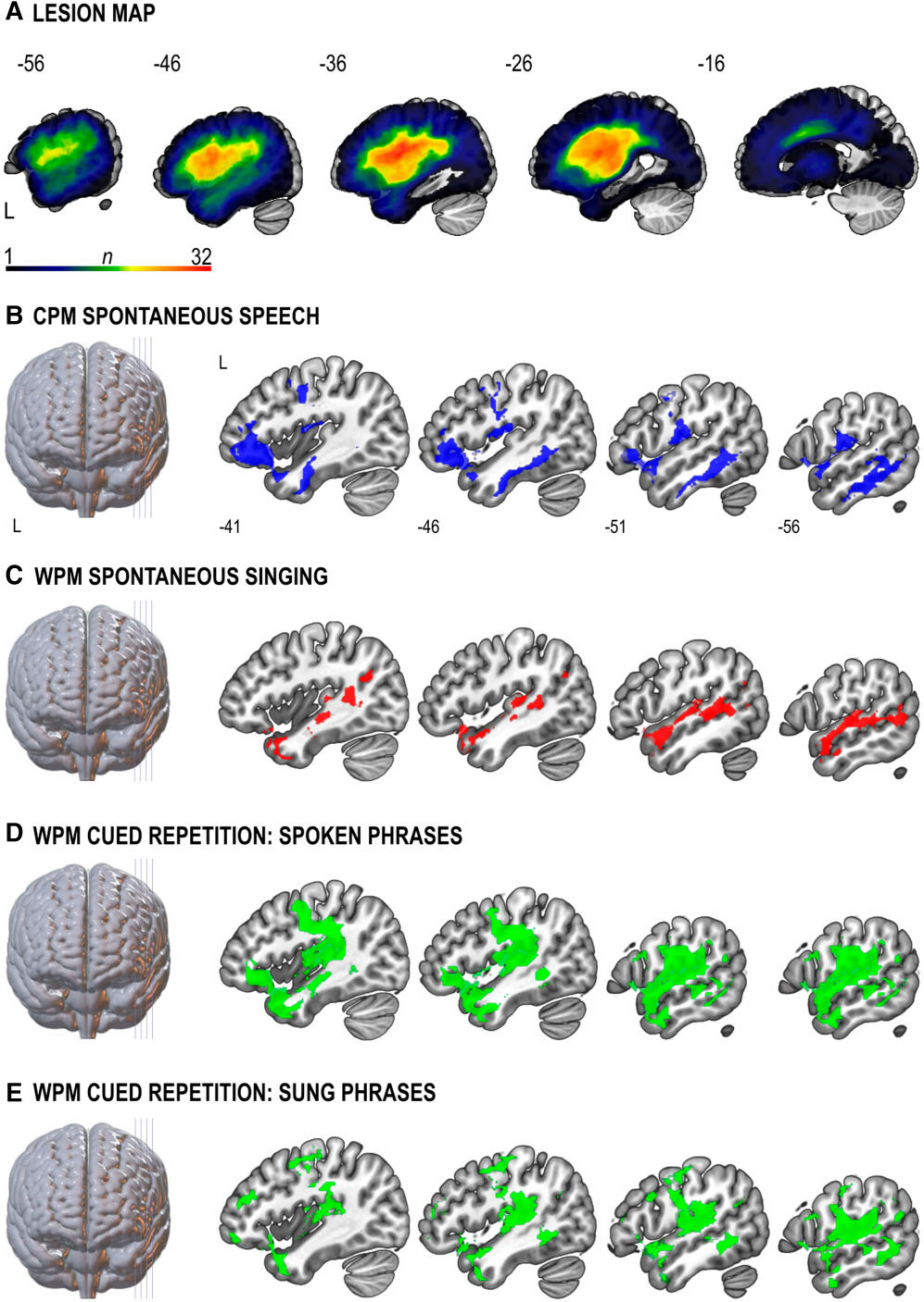
**Lesion patterns associated with dysfluent speech and singing (SVR-LSM)**. (**A**) Lesion overlap map of all patients (*N *= 45). The *n*-value denotes the number of patients with a lesion in each voxel. The greatest lesion overlap among the patients (*n *= 32) was in the vicinity of the left superior longitudinal fasciculus (Montreal Neurological Institute co-ordinate: −32, −6, 22). (**B–E**) Lesion patterns associated with poor performance in (**B**) CPM spontaneous speech production (*n *= 45, cluster *P *= 0.001), (**C**) WPM spontaneous singing (*n *= 43, cluster *P *= 0.002), WPM cued repetition of spoken (*n *= 44, cluster *P* = 0.001) (**D**) and sung (*n *= 44, cluster *P *= 0.002) (**E**) phrases task. All statistical maps are thresholded at cluster-level FWE *P* < 0.05 and adjusted for age and lesion size. CPM, correct information units per minute; L, left; WPM, correct words per minute. Numbers denote Montreal Neurological Institute co-ordinates. See Supplementary Table 3 for further details on anatomical regions. See also Supplementary Figures 1 and 2.

Forty-five chronic stroke patients (25 females, mean age = 64.4 ± 10.2 years) were recruited from Helsinki and Turku regions. Inclusion criteria were time since stroke ≥ 6 months; at least minor non-fluent aphasia due to stroke assessed by Boston Diagnostic Aphasia Examination (BDAE) Aphasia Severity Rating (severity score: 1–5; score  ≤ 4); age ≥ 18 years; Finnish-speaking; no hearing deficit or severe cognitive impairment and no neurological/psychiatric co-morbidity. Patients were defined to have a severe cognitive impairment (e.g. memory disorder or perceptual deficit) if it interfered with their ability to co-operate and provide an informed consent. The study was approved by the Ethics Committees of the Hospital Districts of Helsinki-Uusimaa and Southwest Finland. All patients gave informed consent according to the Declaration of Helsinki. Demographic, musical, clinical and task performance data for all patients are summarized in Table 1 and the lesion map in Fig. 1A. In addition, 34 demographically matched healthy controls were recruited (Supplementary Table 1). In the present study, the fluency dimension was defined on the basis of a defective speech production with a relative preservation of comprehension abilities as reported by the neuropsychologists and speech pathologists with expertise in aphasia. Spontaneous speech, repetition and naming indices of the Western Aphasia Battery (WAB)^26^ were used to evaluate general speech production abilities.

**Table 1:** Summary of demographic, musical, clinical, WAB-R indices and task performance data for all chronic stroke patients with aphasia (*N *= 45)

Demographic information	
Age (years)	64.4 (10.2)
Sex (female/male)	25/20
Handedness (right/left/both)	39/5/1
Education (years)	14.3 (4.1)
Musical background	
Choir singing (years)	3.6 (9.4)
MBEA (rhythm and scale)	22.7 (4.2)
Clinical information	
Lesion size (cm^3^)	95.8 (87.6)
Lesion laterality (left/bilateral)	34/11
Time since stroke (years)	9.0 (7.7)
BDAE severity score	3.0 (1.4)
WAB indices	
Spontaneous speech (max. score 20)	12.9 (7.1)
Repetition (max. score 10)	6.5 (3.5)
Naming (max. score 10)	6.2 (3.6)
Spontaneous speech (CPM)	
Question (Sunday) + Picture description (argument and picnic tasks)	26.3 (21.3)
Spontaneous singing (WPM)	
Spontaneous singing	42.0 (28.8)
Cued repetition (WPM)	
Spoken phrases	46.0 (27.1)
Sung phrases	23.9 (14.5)

Mean (SD) data are presented unless otherwise indicated. BDAE, Boston Diagnostic Aphasia Examination; CPM, correct information units per minute; MBEA, Montreal Battery of Evaluation of Amusia; WAB, Western Aphasia Battery; WPM = correct words per minute.

### Behavioural assessment

Spontaneous vocal production was assessed in the speech domain using the picture description task of the WAB^26^ and personal information and picture sequence description tasks^27^ (three scores averaged together) and in the singing domain by asking the patients to sing (with lyrics) the Finnish version of ‘Brother John’, a well-known nursery rhyme. Given the lack of a standardized protocol for evaluating singing abilities in post-stroke aphasia, this song was selected due to its great popularity and simple melodic and rhythmic structure to maximize word retrieval even in the patients with more severe aphasia, thus making it more comparable with spontaneous speech. Cued repetition was assessed with two tasks where the patients were asked to repeat 16 short phrases presented with normal prosody (speech repetition) and melodic intoning (singing repetition). In each task, the total production time from onset (duration) and number of correct information units (CIUs; in spontaneous speech)^27^ or words (in spontaneous singing and repetition tasks) were first determined and CIUs per minute (CPM) or words per minute (WPM) were then calculated to obtain comparable measures of production rate.^25^ Finally, based on their WPMs in spontaneous singing, the patients were classified into fluent (*N *= 23) and dysfluent (*N *= 20) singers using a normative cut-off value (1.5 SD below the mean value) from the healthy controls in the same task. Only patients with complete behavioural measurements were included in the analyses (spontaneous speech, *N* = 45; spontaneous singing, *N* = 43; cued repetition, *N* = 44) (see Supplementary Methods for details).

### MRI data acquisition and preprocessing

For each patient, a high-resolution T_1_-weighted magnetization prepared rapid gradient echo sequence was acquired using a 3 T Siemens Skyra scanner [repetition time (TR) = 1800 ms; echo time (TE) = 2.27 ms; matrix = 256 × 256; voxel size = 1  mm  × 0.98  mm  × 0.98 mm]. MRI data were preprocessed using the Statistical Parametric Mapping software (SPM12, www.fil.ion.ucl.ac.uk/spm/) under MATLAB R2018b. Stroke lesions were manually delineated to the individual T1 images slice-by-slice by N.M.-M. and A.S. using the MRIcron software package (http://people.cas.sc.edu/rorden/mricron/index.html) and verified by a neuroradiologist (J.P.). Individual T1 and lesion images were reoriented according to the anterior commissure and segmented using Unified Segmentation^28^ with medium regularization and SPM12 IXI data set tissue probability maps. Individual lesion maps were used to apply cost function masking during the preprocessing to achieve optimal normalization of the lesioned brain tissue, with no post-registration lesion shrinkage or out-of-brain distortion.^29^ Due to large lesion sizes, damaged voxels were masked out to achieve an accurate segmentation and spatial normalization. The segmented GM tissue probability maps were modulated and normalized to Montreal Neurological Institute (MNI) space, together with the binarized lesion tracings. Finally, the binary lesion images created in native space were also registered to MNI space and GM and lesion images were smoothed with a 6 mm full width at the half maximum kernel.

### Statistical analysis

Lesion-symptom relations were evaluated using the SVR-LSM.^30^ Four separate SVR-LSM analyses were carried out to identify lesions associated with the four verbal production rates. Only voxels lesioned in at least 10% of the patients were included. Analyses were adjusted for age and lesion volume using a direct total lesion volume control procedure. A voxelwise thresholding was applied after generating SVR β-maps with 10 000 permutations [*P* < 0.005, one-tailed (negative)]. In addition, a cluster size threshold [family-wise error (FWE) rate; *P* < 0.05] was applied for multiple comparisons correction. See Supplementary Methods for details.

In VBM, preprocessed GM images were submitted to four separate multiple regression analyses with CPM or WPM in each task as an explanatory factor. In addition, for patients classified as fluent or dysfluent singers, multiple regression analyses were performed with WPM in spontaneous singing. All analyses were adjusted for age and total intracranial volume. All VBM results were thresholded at FWE cluster-level correction with *P* < 0.05.

Finally, a stepwise regression was performed with WPM in spontaneous singing as the dependent variable to determine the most important predictors for spontaneous singing in aphasia. Age, sex, handedness, education, years of choir singing, Montreal Battery of Evaluation of Amusia, BDAE severity rating, time since stroke, lesion size and lesion laterality as well as the percentage of damage in each patient to 23 GM regions (Supplementary Table 2) involved in vocal motor production (singing and speech) were included as independent variables. Independent sample *T*-tests were run to compare the percentage of damage in these regions between fluent and dysfluent singers and the *P*-values were adjusted using false discovery rate correction to account for the number of multiple comparisons.^31^ See Supplementary Methods for details.

### Data availability

The data that support these findings are available from the corresponding author, upon reasonable request.

## Results

### Lesion patterns linked to speech and singing in aphasia: multivariate SVR-LSM

First, we examined the relationship between lesion distribution and speech and singing production (Fig. 1 and Supplementary Table 3). Poor spontaneous speech was associated with lesions in the left inferior frontal gyrus (IFG) and middle frontal gyrus (MFG), superior temporal pole (TPO-STG), Rolandic operculum (ROL), inferior temporal gyrus and insula (Fig. 1B). Lesions linked to poor spontaneous singing were located exclusively in left temporal regions: middle temporal gyrus (MTG) and superior temporal gyrus (STG), middle temporal pole (TPO-MTG) and TPO-STG (Fig. 1C). Lesions associated with poor cued repetition of speech and singing comprised the left frontal (IFG) and temporal (Heschl’s gyrus, STG, MTG, TPO-STG, TPO-MTG and ROL) areas (Fig. 1D and E). Beyond this shared lesion pattern, lesions in the left MFG were linked to poor singing repetition.

Next, we evaluated how well damage in areas within the vocal motor network predicted poor performance in the singing task compared with stroke-related demographic and clinical variables. The stepwise regression showed that greater damage in the left TPO-STG was the only predictor of poor spontaneous singing [*R*^2 ^= 0.233, *β* = −49.36, *F*(1,39)_ _= 11.86, *P *< 0.001].

### GMV supporting speech and singing in aphasia: VBM

**Figure 2:**
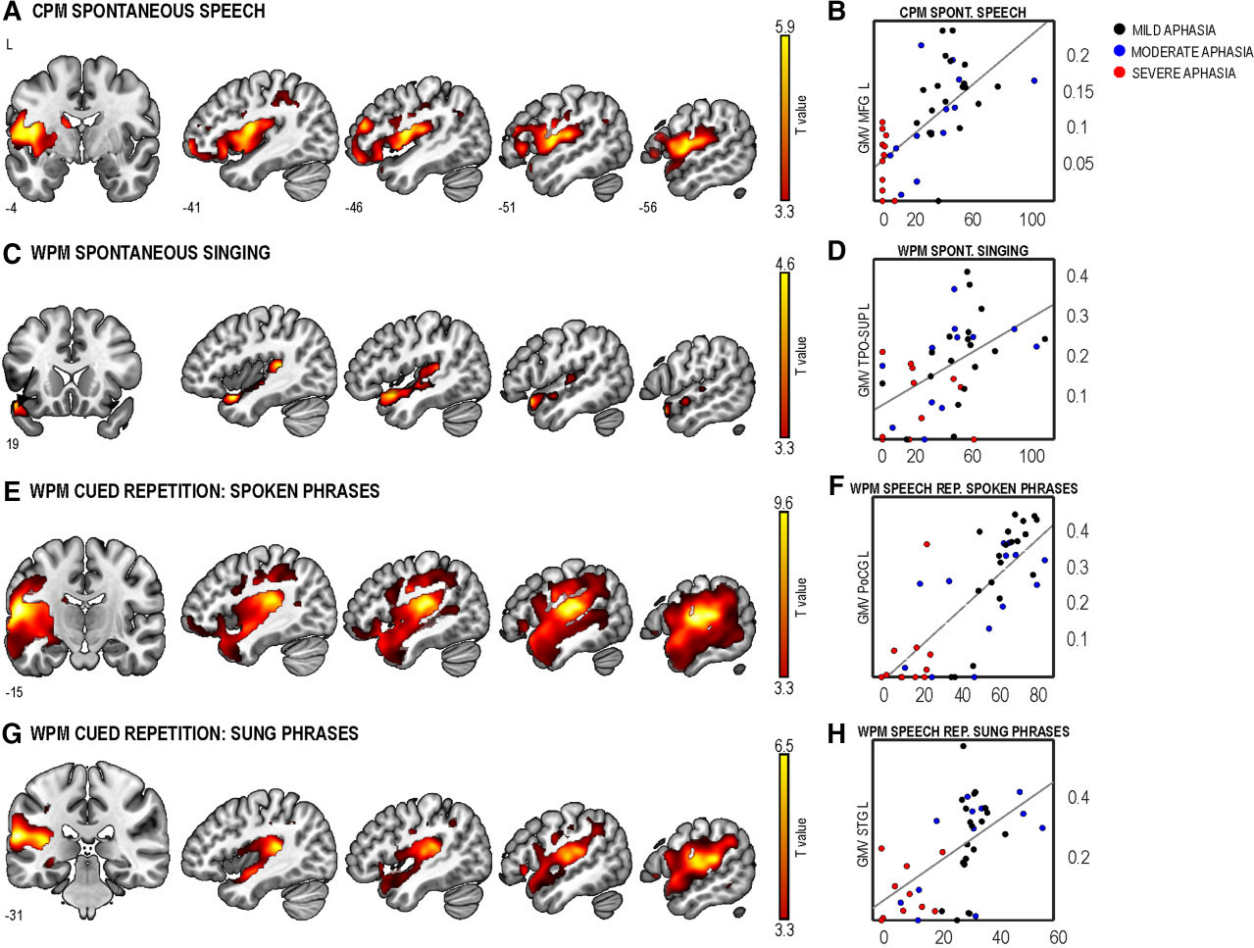
**GMV positively correlated with production in the four tasks**. (**A**) CPM in spontaneous speech [*n* = 45, cluster-level *P* < 0.001; *T*(1,41)_ _= 5.889]. WPM in spontaneous singing [*n* = 43, cluster-level *P* < 0.001; *T*(1,39)_ _= 4.558] (**C**), cued repetition of spoken phrases [*n* = 44, cluster-level *P* = 0.000; *T*(1,40)_ _= 9.641] (**E**), cued repetition of sung phrases [*n* = 44, cluster-level *P* = 0.000; *T*(1,40)_ _= 6.549] (**G**). All statistical maps are thresholded at cluster-level FWE *P* < 0.05 and adjusted for age and total intracranial volume. (**B, D, F and H**) Scatter plots showing the correlation between GMV for the clusters defined as the voxel with the highest *T*-value in the multiple regression analyses and production rates (WPM, CPM) of the corresponding task. GMV in the scatter plots represent the patient’s value from the Montreal Neurological Institute co-ordinate with the highest *T*-value for each FWE-corrected cluster. Datapoints from patients with mild (BDAE = 4–5), moderate (BDAE = 3) or severe aphasia (BDAE = 1–2) are depicted in black, blue and red colours, respectively. BDAE, Boston Diagnostic Aphasia Examination; CPM, correct information units per minute; L, left; MFG, middle frontal gyrus; PoCG, postcentral gyrus; STG, superior temporal gyrus; TPO-SUP, superior temporal pole; WPM, correct words per minute. Numbers denote Montreal Neurological Institute co-ordinates. Colour bars indicate *T*-values.

Better spontaneous speech was associated with greater GMV in the left MFG (Fig. 2A and B and Supplementary Table 4). In turn, better spontaneous singing was associated with greater GMV in the left STG including the TPO-STG (Fig. 2C and D and Supplementary Table 5). Better cued repetition of spoken and sung phrases was associated with greater GMV in the left post-central gyrus (PoCG) and STG, respectively (Fig. 2E–F and G–H and Supplementary Tables 6 and 7).

### Relationship between GMV and fluent or dysfluent singing

**Figure 3:**
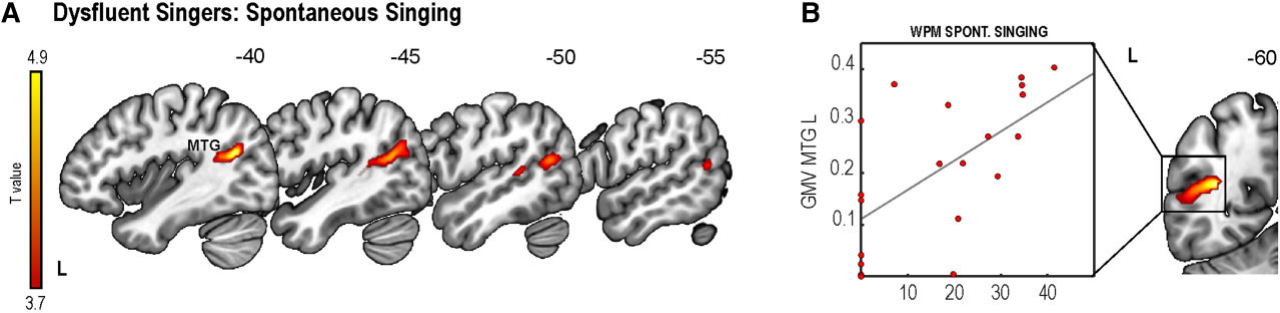
**GMV in patients classified as dysfluent singers based on a singing production cut-off from healthy participants.** (**A**) GMV associated with WPM spontaneous singing of a familiar song in dysfluent singers [*n* = 20, cluster-level *P* = 0.006; *T*(1,16)_ _= 4.933]. (**B**) Scatter plot showing the correlation between GMV for the cluster defined as the voxel with the highest *T*-value in the multiple regression analysis and spontaneous singing. GMV in the scatter plot represent the patient’s value from the Montreal Neurological Institute co-ordinate with the highest *T*-value for the FWE-corrected cluster. L, left; MTG, middle temporal gyrus. Numbers denote Montreal Neurological Institute co-ordinates. Colour bar indicates *T*-values.

Crucially, there were no significant differences in demographic, clinical and musical information or behavioural performance between the fluent and dysfluent singers (Supplementary Table 8). In dysfluent singers, a positive relationship between GMV and performance in spontaneous singing was found in the left MTG (Fig. 3A and B and Supplementary Table 9). In fluent singers or healthy controls, no significant relationships were found. To further clarify the relationship between singing production and lesion load, we compared the percentage of damage in the vocal motor network between fluent and dysfluent singers. This comparison demonstrated that only the temporal regions in the left hemisphere were significantly more damaged in the group of dysfluent singers (Supplementary Table 10).

## Discussion

In this study, we report the neuroanatomical correlates of speech and singing production in chronic aphasia. Our results revealed lesion patterns for connected speech and cued repetition overlapping with the left-hemispheric speech production network.^32^ Spontaneous singing was associated with damage to the left STG and MTG. Furthermore, damage to the left TPO-STG was the sole predictor of task performance, beyond clinical variables, such as aphasia severity, as well as damage to frontoparietal regions within the traditional speech production network. The VBM results confirmed that preservation of GMV in the same regions where damage led to poor speech and singing production supported better performance in these tasks. Finally, within the dysfluent singer group, preservation of left posterior MTG was related to better spontaneous singing, and lesion differences between the dysfluent and fluent groups were focalized to the left STG and MTG.

For spontaneous speech production, we used a composite score from a direct question and two picture description tasks. Our SVR-LSM findings converge with previous studies using similar tasks, which have shown that damage to an extensive left frontotemporoparietal network impairs connected speech in chronic stroke patients.^15,23,33^ Our findings are also consistent with recent unified models of post-stroke aphasia at the chronic stage.^34^ Speech rate, which in this study reflects the speed as well as accuracy of the information produced, encompasses multiple cognitive processes, from retrieval of correct words to form syntactic structures to phonological planning and articulation.^35^ Consequently, the lesion patterns we observed here overlapped with brain regions associated with these components^34^ and mapped into the dorsal and ventral streams for speech production.^32^

Performance in spontaneous singing was associated with the left STG (SVR-LSM and VBM) and MTG (SVR-LSM). This aligns with previous fMRI evidence in healthy participants^7^ and training-related GMV differences in professional singers.^12^ Our findings suggest that the sparing of temporal regions in singing might be crucial to bypass the dorsal high-level circuit for speech production involving motor maps coded in the left IFG, which is commonly damaged in non-fluent aphasia, by recruiting a sensorimotor network for vocal production where auditory targets interact with articulatory motor programmes in the primary motor cortex.^36^ The extent to which this pattern of results applies to non-fluency due to aphasia and/or apraxia of speech (AOS) remains an open question. Future research including a specific battery for evaluating AOS should allow us to examine the relationship between AOS after stroke and spontaneous singing performance.

For speech and singing repetition, the anatomical distribution of the lesion patterns greatly overlapped with each other and with spontaneous speech. Interestingly, the lesion pattern for speech repetition was larger and included a greater portion of the left IFG (Fig. 1D), further supporting the notion that, as opposed to singing, speech may be more affected by damage in this area. The left MFG was linked to poor singing repetition. Lesions in this region, which is involved in executive processes including monitoring of self-performance,^37^ could interfere with the increased performance monitoring demand of repeated singing compared to repeated speech. Regarding the VBM results, it is also worth noting that the global maxima in the cluster for spontaneous speech was located in frontal regions whereas it was more posteriorly distributed in the cued repetition of speech (ventral PoCG) and singing (STG). This indicates that performance in the repetition of formulaic phrases, which likely relies on preserved motor automaticity, may be mainly associated with lesions in the areas implicated in phonological production.^35,36^

Our structural findings for singing production could be integrated within two influential neuroanatomical models of speech motor control: the directions into velocity of articulators (DIVA)^38^ and hierarchical state feedback control (HSFC)^36^ models. Based on the SVR-LSM and VBM clusters showing an anterior–posterior distribution along the STG for spontaneous singing in chronic aphasia, one plausible underlying mechanism could be that auditory targets for singing stored in the posterior STG (pSTG) would interact with speech motor maps to select the corresponding articulatory programmes (as posited by DIVA) if the Spt, a region in the Sylvian fissure involved in auditory-motor integration, is intact (as postulated by HSFC). This account is consistent with volumetric changes that have been found in association with singing training.^12^ Our VBM findings in dysfluent singers as well as previous volumetric changes in the STG/MTG and cerebellum found in aphasic patients for sung versus spoken verbal memory^5^ also lend support to this interpretation. In addition, an fMRI case study reported increased left pSTG and pre/post-central activity induced by MIT in a patient with chronic Broca’s aphasia.^17^

Alternatively, left TPO-STG could play an important role before co-ordinate transformation of the auditory-motor maps in the Spt. This interpretation is in agreement with our regression analysis results where damage to the left TPO-STG was the strongest predictor of spontaneous singing performance, beyond stroke-related variables. Previous fMRI studies have also shown increased engagement of anterior temporal regions when listening to well-known tunes both in healthy and patient groups^39,40^ as well as greater activation by singing versus speech perception in these regions.^10^ Moreover, anterior temporal regions have been linked to better learning and recognition of sung verbal material after stroke^4^ or epilepsy surgery.^41^ Beyond these two accounts, the vocal motor maps for singing could be stored in the right hemisphere, as it is more activated during covert singing than covert speech,^7^ and future fMRI studies could directly address this question. Although the explanations put forward should be regarded as tentative, it is clear that preservation of left temporal regions seems crucial for fluent spontaneous singing in chronic aphasia.

Regarding the laterality of our results, all the GMV clusters associated with better singing performance were located in the left hemisphere while none were observed in the right hemisphere. At first, these findings may appear somewhat paradoxical as previous studies have provided evidence that frontotemporal and parietal regions or pathways in both hemispheres (i) are activated by the perception and production of singing,^7–10^ (ii) show structural/functional neuroplasticity changes induced by singing training^11–13,42,43^ in the healthy brain and (iii) are linked to the efficacy of music-based aphasia rehabilitation that involves singing-based training^15–19,44^ or daily listening to vocal music.^21,22^ The lack of VBM clusters in the right hemisphere may be driven by the fact that our behavioural outcome measure in all tasks was the efficiency of the verbal (linguistic) output of the patients, which in the case of a familiar song requires semantic processing and memory retrieval known to be supported primarily by the left ventral stream structures.^45^ It is possible that the non-verbal (musical) perceptual and expressive aspects of singing, such as pitch and melody, which tend to show rightward lateralization in the healthy brain,^42,46–48^ may be more closely linked to right hemispheric structures also in aphasia. It is also possible that other neuroimaging measures that look at structural connectivity (diffusion tensor imagin tractography), functional connectivity (network analysis of resting-state fMRI) or functional activation (fMRI singing production task) could show the functional role of the right hemisphere on singing in aphasia, which the structural regional measures used in the present study are not able to capture. These issues clearly need to be addressed in future studies.

In conclusion, this novel study provides the first evidence on the neuroanatomical correlates of singing in chronic aphasia and corroborates previous findings on speech production. Ultimately, these results may guide the stratification of patients for future longitudinal studies with singing-based interventions for speech recovery and provide putative biomarkers of treatment response.

## Supplementary Material

fcac001_Supplementary_Data

## Abbreviations

BDAE =: Boston Diagnostic Aphasia Examination
SVR-LSM =: support vector regression-based lesion-symptom mapping
VBM =: voxel-based morphometry
WAB =: Western Aphasia Battery
