# Correction to: Neuroanatomical correlates of speech and singing production in chronic post-stroke aphasia

**DOI:** 10.1093/braincomms/fcac265

**Published:** 2022-10-21

This is a correction to: Noelia Martínez-Molina, Sini-Tuuli Siponkoski, Anni Pitkäniemi, Nella Moisseinen, Linda Kuusela, Johanna Pekkola, Sari Laitinen, Essi-Reetta Särkämö, Susanna Melkas, Boris Kleber, Gottfried Schlaug, Aleksi Sihvonen, Teppo Särkämö, Neuroanatomical correlates of speech and singing production in chronic post-stroke aphasia, *Brain Communications*, Volume 4, Issue 1, 2022, fcac001, https://doi.org/10.1093/braincomms/fcac001

In the originally published version of this manuscript, in Table I, the row “Spontaneous speech (CPM): Question (Sunday) + Picture description” featured incorrect value of 26.3(21.3). The correct value is 30.8(25.5).

This error has been corrected.

