# Supplementary material for: Neuroanatomical correlates of speech and singing production in chronic post-stroke aphasia
Source: Brain Commun. 2022 Jan 11;4(1):fcac001. doi: 10.1093/braincomms/fcac001 (PMC8842683; doi:10.1093/braincomms/fcac001)
Supplement: fcac001_Supplementary_Data [file fcac001_Supplementary_Data.docx]

**SUPPLEMENTARY METHODS**

**Behavioural measures**

The behavioural tasks were completed during a 2-hour neuropsychological baseline assessment, which was part of a larger intervention study. The measurements took place at the University of Helsinki (Helsinki) and Rehabilitation Center Suvituuli (Turku) in a quiet space and they were conducted by a trained psychologist. The subjects had a possibility for a short break during the examination if needed. The aphasia severity rating (*Boston Diagnostic Aphasia Examination, BDAE*)^1^ was determined by the clinical psychologist based on the observations during this assessment. Spontaneous speech, Repetition and Naming indices of the *Western Aphasia Battery (WAB)*^3^ were used to evaluate general speech production abilities. All the computer-based tasks were conducted in Presentation ([https://www.neurobs.com/)*.*](https://www.neurobs.com/).%20) The Praat software ([https://www.fon.hum.uva.nl/praat/)](https://www.fon.hum.uva.nl/praat/)%20) was used for analysing the recordings. In addition, the aphasia severity rating *(Boston Diagnostic Aphasia Examination, BDAE)* ^1^, which had been initially estimated in the recruitment interview, was verified by the clinical psychologist based on the observations during this assessment.

**Spontaneous speech production.** The spontaneous speech production was assessed using three separate tasks: (i) Personal information (*Tell me what you usually do on Sundays*)^2^; (ii) picture description task (*Picnic*) from WAB^3^; and (iii) Sequential picture description task (*Argument)*^2^. The spontaneous speech production tasks were presented consecutively in this order and the productions were recorded. Patients were encouraged to speak for as long as possible. The speech production data was analysed by determining the time of the total production, as measured from the onset to the end of speech production related to the subject using Praat Annotation tools. Comments clearly not related to the question were elicited from the beginning and the end of the production, additionally the prompts or questions provided by the researcher were subtracted from the speaking time. The amounts of words and correct information units (CIUs) were calculated, following the guidelines by Nicholas and Brookshire^2^. These values were then used to calculate the words and CIUs per minute (CPM) in each task. The analysis of spontaneous speech was conducted by a neuropsychologist (S.-T.S.). For analytical purposes we used a composite index of spontaneous speech production by averaging the CPMs in each task.

**Spontaneous singing production.** The singing task (Finnish version of a well-known nursery rhyme: ‘Brother John’) was computerized and consisted of singing the song spontaneously with lyrics. The productions were analysed by calculating: (i) the amount of recognisable words (including also words with minor phonemic distortions as correct units, in order to make the rating comparable with the spontaneous speech production); and (ii) duration of the production, starting from the onset of production.

| **Jaakko-kulta lyrics (Finnish version)** | **Brother John Lyrics** |
| --- | --- |
| *Jaakko-kulta*  *Jaakko-kulta*  *Herää jo*  *Herää jo*  *Kellojasi soita*  *Kellojasi soita*  *pium, paum, poum*  *pium, paum, poum* | Brother John  Brother John Are you sleeping  Are you sleeping  Morning bells are ringing Morning bells are ringing ding, dang, dong,  ding, dang, dong |

**Cued Repetition.** The subjects were presented 16 phrases, which were progressive in length (1-5 words). Altogether the phrases included 42 words and were designed according to melodic intonation therapy principles^4^. First, the same phrases were presented in spoken and then in sung format and the subjects were asked to repeat them accordingly. The models were recorded by a music therapist (S.L.). The sung phrases were presented in a melodic intonation therapy –type of intoning. The lengths of the models for spoken phrases varied between 1.07 seconds and 3.95 seconds, and the lengths of the models for sung phrases between 2.02 seconds and 7.90 seconds. During the task, subjects had the possibility to ask for repetition when they clearly could not repeat the phrase at first attempt; however, this was merely to ease the stress and support motivation, since only first productions were analysed. The productions were analysed by calculating: (i) the amount of recognisable words (including words with minor distortions as correct units); and (ii) the length of the production, starting from the onset. Everything, except clearly unrelated material (*e.g. the subject telling that he/she is not able to repeat or starting to speak about another topic*) were included in the estimated production length.

| **Original phrase (Finnish)** | **English translation (free)** |
| --- | --- |
| *Huomenta*  *Nähdään taas*  *Laulan mielelläni*  *Oletko pettynyt?*  *Hanki on valkoinen*  *Lepään viikonloppuna*  *Tule syömään lounasta*  *Elämän nälkä eteenpäin rohkaisee*  *Näkemiin*  *Hyvää yötä*  *Pidän musiikista*  *Oletko vihainen*  *Taivas on sininen*  *Istu tähän vierelleni*  *Huomenna tulee vieraita*  *Ylös kengät paina ei jalkaa* | Good morning  See you again  I like to sing  Are you disappointed?  The snow is white  I rest during the weekend  Come and have lunch  Hunger for life pushes you on  Good bye  Good night  I like music  Are you angry?  The sky is blue  Come sit with me  Guests are coming tomorrow  Get up, shoes don’t weigh you down |

Only patients with complete recordings in each of the tasks were considered for the analyses: spontaneous speech (N=45), singing task (spontaneous: N=43), spoken and sung repetition (N=44). See Supplementary Figures S1-2 for the lesion map of these subset of patients.

**Imaging acquisition**

Patients completed a scanning session in the HUS Helsinki Medical Imaging Center at Helsinki University Central Hospital and in the Department of Radiology at Turku University Hospital. Images were acquired on a 3T Siemens Skyra scanner using a 64-channel head coil. For each patient, a high-resolution T1-weighted magnetization prepared rapid gradient echo (MPRAGE) sequence was acquired with the following parameters: 192 sagittal slices; slice thickness = 1 mm, field of view (FOV) = 261 x 261 mm; matrix = 256 x 256; repetition time (TR) = 1800 ms; echo time (TE) = 2.27 ms; inversion time (TI) = 900 ms; flip angle = 8°, voxel size = 1 x 0.98 x 0.98 mm. Fluid-attenuated inversion recovery (FLAIR) images were also obtained to confirm lesion location, size and shape on a different MRI modality. The parameters for this scan included: 192 sagittal slices; slice thickness = 0.9 mm, field of view (FOV) = 261 x 261 mm; matrix = 256 x 256; repetition time (TR) = 5000 ms; echo time (TE) = 398 s; inversion time (TI) = 1800 s; flip angle = 120°, voxel size = 0.94 x 0.94 x 1.05 mm.

**Lesion tracing and normalization**

The post-stroke chronic lesion tracing images were manually delineated using the drawing tools in MRIcron v1.0.20190902 (<https://www.nitrc.org/projects/mricron>) on the T1-weighted images by N.M.M. and A.S, who were blind to the behavioural scores at the time of the lesion drawing. FLAIR images were referenced for additional confirmation of lesion location, size and shape. For patients with bilateral lesions, the lesion in the right hemisphere was also delineated and included in the computation of lesion size. The lesion tracing images (binarized) were visually inspected by a board-certified radiologist (J.P.) to verify drawing accuracy. Hypointensities due to small lacunar strokes were not included in these lesion masks. The lesion overlap maps are shown in Figure 1 and Supplementary Figures S1-2. The lesion tracings were spatially normalized to MNI space using unified segmentation and normalization procedures in SPM12 (<https://www.fil.ion.ucl.ac.uk/spm/software/spm12>) by applying the spatial normalization parameters derived from the segmentation of T1-weighted images after masking out the lesion tracings as described in the VBM protocol. The tissue probability maps in SPM12 from the IXI dataset including more than 600 subjects were used for segmentation. The resliced 2 mm^3^ lesion masks were then smoothed with a 6 mm full-width at half-maximum Gaussian kernel to remove uneven edges associated with manual drawing.

**Multivariate SVR-LSM**

We applied a multivariate approach called support vector regression-based lesion symptom-mapping (SVR-LSM)^5^ to identify brain areas implicated in spontaneous speech production (personal question, picture description), spontaneous singing and phrase repetition (spoken and sung format) processes. Given the nonrandom nature of lesion distribution and the resultant autocorrelation of voxel lesion status, univariate methods such as voxel-based LSM (VLSM)^6^ are vulnerable to mislocalization, especially when multiple brain areas support the cognitive process under study^7^. Here, consideration of lesion covariance is of importance since speech and music processing including singing rely on a distributed network of coordinated regions^8, 9^. SVR-LSM considers the lesion status of all voxels in a single regression model and is less vulnerable to lesion mislocalization and more sensitive to nonlinear relationships. We applied SVR-LSM in MATLAB R2018b via a graphical user interface implementation developed by DeMarco and Turkeltaub^10^ (<https://github.com/atdemarco/svrlsmgui/>). Only voxels lesioned in at least 10% of participants were included in each analysis. We controlled for effects of lesion volume using direct total lesion volume control (dTLVC), a method whereby the voxel values in each lesion map are divided by the square root of the lesion volume for that patient.

Four one-tailed (negative) SVR-LSM analyses were run to identify lesions associated with words per minute in: (i) spontaneous speech, (ii) singing from memory, and repetition of phrases in (iii) spoken and (iv) sung format as the dependent variable in each of these analyses. Age was covaried with the lesion data to remove nuisance effects. The analysis was run with parameters gamma = 5 and cost = 30 as reported in the literature^10^. Significance was determined using a permutation-based approach in which the behavioral scores were randomly reassigned to participants and SVR-β-value maps were generated for each of 10,000 permutations.

SVR-β values were catalogued on a voxelwise basis and thresholded at *P* < 0.005 (one-tailed). To correct these maps for multiple comparisons, a cluster size threshold was applied to achieve a familywise error rate of 0.05 based on the largest cluster in each of the voxelwise thresholded permutation maps. This permutation approach minimizes the effects of lesion autocorrelation in LSM analyses^11^.

**VBM protocol**

VBM is a whole-brain technique allowing the identification of local changes in grey matter density or volume by applying voxel-wise statistics within the context of Gaussian random fields^12^. The preprocessing and analyses were performed in SPM12 running under Matlab R2018b. Prior to preprocessing, all images were manually reoriented to the anterior commissure to reduce between-subject variability and facilitate registration with the tissue probability maps. Lesion masks were applied to mask out damaged voxels to achieve accurate segmentation and spatial normalization. Data were subsequently processed via a procedure of joint spatial normalization and segmentation using the unified segmentation approach^13^ with the tissue probability maps in SPM12 from the IXI dataset including more than 600 subjects. Specifically, images were corrected for bias-field inhomogeneity and segmented into grey matter, white matter and CSF maps using light cleanup and medium regularisation. The segmented maps were then registered to a standard template in MNI space using the 12-parameter affine linear and non-linear warping transformation. Grey matter voxel values were then multiplied by the Jacobian matrix parameters derived from normalisation to preserve original grey mater values locally (modulated grey matter volumes.) The modulated grey matter volumes were then smoothed with a Gaussian kernel of 6 mm full-width at half-maximum to reduce anatomical variability.

**Fluent and dysfluent singers’ classification**

We determined the words per minute production rate of spontaneous singing of Jaakko-kulta in healthy older adults (N = 34) matched to the stroke patients in demographic and musical background (Table S1). The mean of the control group (M = 60.32) minus 1.5 standard deviation (SD = 9.46) was used as the lower threshold for singing fluency (patients above that cutoff value were classified as fluent singers). Crucially, there were no significant differences in demographic, clinical and musical information or behavioural performance between the fluent (n=23) and dysfluent (n=20) singers (Table S8). Note that two patients with no recordings from spontaneous singing were excluded from this analysis.

**Lesion load**

Once the lesion masks were normalized to MNI space, they were converted to .mat files and overlaid to the automated anatomical atlas^14^ regions using Matlab scripts from the NiiStat toolbox (<https://github.com/neurolabusc/NiiStat>). The percent of stroke-related damage within the 23 grey matter regions for each individual patient was then saved for statistical analyses. We were interested in including frontal and temporal regions relevant for vocal production based on the existing literature on speech and singing production (see Table S2).

**Statistical analyses**

A stepwise regression was performed with WPM in spontaneous singing as dependent variable. Age, gender, handedness, years of education, years of singing, MBEA, BDAE, time since stroke, lesion size and lesion laterality as well as the percentage of damage in each patient to 23 cortical regions involved in singing production were included as independent variables. The entry criterion was *P* < 0.05, and the exit criterion was *P* > 0.10. Tests for multicollinearity indicated that a very low level of multicollinearity was present in the analysis (variance inflation factors < 1.09 and tolerances > 0.9). Independent samples T-tests were run to compare this percentage of damage between the fluent and dysfluent singers. FDR with the Benjamini & Hochberg method^15^ was applied to correct for multiple comparisons.

**SUPPLEMENTARY FIGURES AND TABLES**


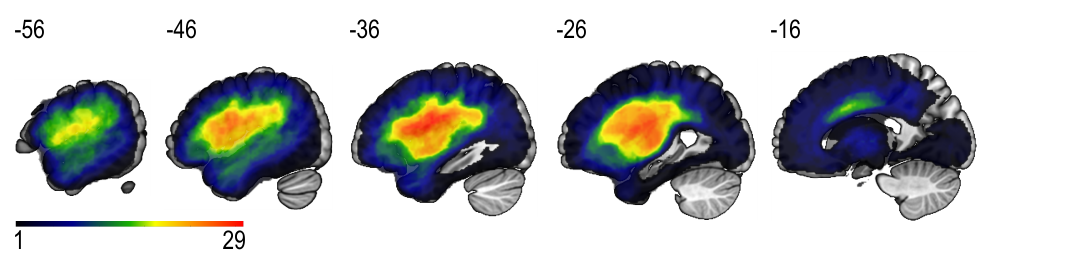


**Supplementary Figure 1.** **Lesion overlap of 43 patients with chronic post-stroke aphasia used in the SVR-LSM analysis with words per minute in spontaneous singing of a familiar song.** The colourbar denotes the number of patients with a lesion in each voxel. The greatest lesion overlap among the patients (n=29) was in the vicinity of the left Superior Longitudinal Fasciculus (MNI coordinate: -32, -6, 22). L: left.


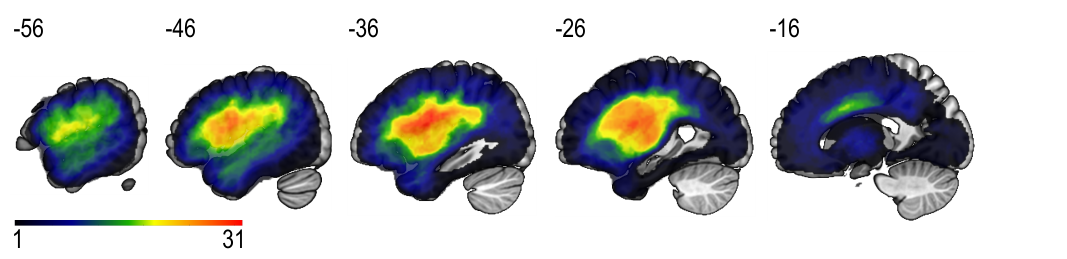


**Supplementary Figure 2**. **Lesion overlap of 44 patients with chronic post-stroke aphasia used in the SVR-LSM analysis with words per minute in MIT with phrases in spoken and sung format.** The colourbar denotes the number of patients with a lesion in each voxel. The greatest lesion overlap among the patients (n=31) was in the vicinity of the left Superior Longitudinal Fasciculus (MNI coordinate: -32, -6, 22). L: left.

**Supplementary Table 1. Demographic and musical background of healthy controls and stroke patients.** Mean (SD) data are presented unless otherwise indicated.

|  | **Healthy controls (N=34)** | **Stroke Patients (N=45)** | **Difference between groups (P)** |
| --- | --- | --- | --- |
| **Demographic information** | | | |
| **Age** (years) | 64.1 (10.3) | 64.4 (10.2) | .901 (t) |
| **Gender** (female / male) | 19 / 15 | 25 / 20 | .977 (χ^2^) |
| **Handedness** (right/left/both) | 34/0/0 | 39 / 5/ 1 | .080 (χ^2^) |
| **Education** (years) | 15.7 (4.8) | 14.3 (4.1) | .178 (t) |
| **Musical background** | | | |
| **Choir singing** (years) | 15.4 (18.3) | 8.0 (17.1) | .070 (t) |

**Supplementary Table 2.** 23 Grey matter regions from the Automated Anatomical Atlas used to determine the percent of stroke related damage in each patient.

| **AAL atlas label**  **(Abbreviated name)** | **AAL atlas label**  **(Full name name)** |
| --- | --- |
| **Precentral_L^16^** | Left precentral gyrus |
| **Frontal_Sup_L^16^** | Left superior frontal gyrus |
| **Frontal_Sup_Orb_L^16^** | Left superior frontal gyrus pars orbitalis |
| **Frontal_Mid_L^16^** | Left middle frontal gyrus |
| **Frontal_Mid_Orb_L^16^** | Left middle frontal gyrus pars orbitalis |
| **Frontal_Inf_Oper_L^16^** | Left inferior frontal gyrus pars opercularis |
| **Frontal_Inf_Tri_L^16^** | Left inferior frontal gyrus pars triangularis |
| **Frontal_Inf_Orb_L^16^** | Left inferior frontal gyrus pars orbitalis |
| **Rolandic_Oper_L^17, 18^** | Left Rolandic operculum |
| **Supp_Motor_Area_L^17^** | Left supplementary motor area |
| **Frontal_Sup_Medial_L^16^** | Left superior frontal gyrus medial |
| **Frontal_Med_Orb_L^16^** | Left medial orbital superior frontal gyrus |
| **Insula_L^17, 18^** | Left insula |
| **Cingulum_Ant_L^17^** | Left anterior cingulate gyrus |
| **Cingulum_Mid_L^17^** | Left middle cingulate gyrus |
| **Cingulum_Post_L^16^** | Left posterior cingulate gyrus |
| **Postcentral_L^17^** | Left postcentral gyrus |
| **Heschl_L^17^** | Left Heschl gyrus |
| **Temporal_Sup_L^16^** | Left superior temporal gyrus |
| **Temporal_Pole_Sup_L^16^** | Left superior temporal pole |
| **Temporal_Mid_L^16^** | Left middle temporal gyrus |
| **Temporal_Pole_Mid_L^16^** | Left middle temporal pole |
| **Temporal_Inf_L^16^** | Left inferior temporal gyrus |

**Supplementary Table 3.** Size and location of SVR-LSM clusters associated with speech and singing production tasks.

| **Task** | **Cluster P value** | **Cluster volume (mm^3^)** | **Center of mass (MNI coordinate)** | **Anatomical region overlap (AAL)** | **Maximal lesion overlap (n)** |
| --- | --- | --- | --- | --- | --- |
| **Spontaneous speech** | 0.001 | 46248 | -41, 8, -5  (Insula L) | Frontal Inf Orb L (56.22%)  Frontal Mid Orb L (37.49%)  Frontal Inf Tri L (26.09%)  Temporal Pole Sup L (25.52%)  Rolandic Oper L (21.24%)  Temporal Inf L (17.72%)  Insula L  (16.79%) | 18 |
| **Spontaneous singing** | 0.002 | 20144 | **-**48, -19, -6  (Temporal Sup L) | Temporal Pole Sup L (30.20%)  Temporal Sup L (30.05%)  Temporal Pole Mid L (18.37%)  Temporal Mid L (14.86%) | 13 |
| **Cued spoken repetition** | 0.001 | 74728 | **-**46, -9, 3  (Rolandic_Oper_L) | Heschl L (82.05%)  Temporal Sup L (59.23%)  Temporal Pole Sup L (57.22%)  Rolandic Oper L (56.04%)  Frontal Inf Orb L (40.47%)  Temporal Pole Mid L (39.13%)  Insula L (27.94%)  Temporal Mid L (23.92%)  Frontal Inf Tri L (12.49%) | 20 |
| **Cued sung repetition** | 0.002 | 43776 | -49, -13 ,7  (Heschl L) | Heschl L (52.41%)  Temporal Sup L (46.85%)  Rolandic Oper L (32.93%)  Temporal Pole Sup L (31.57%)  Frontal_Mid_Orb_L (16.49%)  Frontal_Inf_Orb_L (15.83%)  Temporal_Mid_L (15.23%)  Temporal_Pole_Mid_L (13.22%)  Insula L (10.48%) | 20 |

**Supplementary Table 4. Grey matter volume clusters showing a significant positive correlation with correct information units per minute in spontaneous speech production at cluster-level P (FWE-corrected) < 0.05.** The x,y,z coordinates are according to the MNI atlas (mm). Nuisance covariates included age and total intracranial volume.

| **Anatomical region** | **Cluster size (Voxels)** | **T value** | **Cluster-level P(FWE-corr)** | **x** | **y** | **z** |
| --- | --- | --- | --- | --- | --- | --- |
| Left Frontal Middle Gyrus | 11288 | 5.889 | 0 | -50 | 38 | 20 |
| Left Rolandic Operculum |  |  |  | -44 | -4 | 12 |
| Left Postcentral Gyrus |  |  |  | -54 | -6 | 14 |

**Supplementary Table 5.** **Grey matter volume clusters showing a significant positive correlation with words per minute in spontaneous singing of a familiar song at cluster-level P (FWE-corrected) < 0.05.** The x,y,z coordinates are according to the MNI atlas (mm). Nuisance covariates included age and total intracranial volume.

| **Anatomical region** | **Cluster size (Voxels)** | **T value** | **Cluster-level P(FWE-corr)** | **x** | **y** | **z** |
| --- | --- | --- | --- | --- | --- | --- |
| Left Superior Temporal Pole | 1533 | 4.558 | 2.18 x 10 ^-05^ | -42 | 10 | -18 |
| Left Superior Temporal Gyrus |  |  |  | -42 | -36 | 10 |

**Supplementary Table S6.** **Grey matter volume clusters showing a significant positive correlation with words per minute in speech repetition of phrases in spoken format at** **cluster-level P (FWE-corrected) < 0.05.** The x,y,z coordinates are according to the MNI atlas (mm). Nuisance covariates included age and total intracranial volume. MIT: Melodic Intonation Therapy.

| **Anatomical region** | **Cluster size (Voxels)** | **T value** | **Cluster-level P(FWE-corr)** | **x** | **y** | **z** |
| --- | --- | --- | --- | --- | --- | --- |
| Left Postcentral Gyrus | 21821 | 9.641 | 0 | -58 | -14 | 16 |
| Left Superior Temporal Gyrus |  |  |  | -60 | -32 | 18 |

**Supplementary Table 7.** **Grey matter volume clusters showing a significant positive correlation with words per minute in speech repetition of phrases in sung format at cluster-level P (FWE-corrected) < 0.05.** The x,y,z coordinates are according to the MNI atlas (mm). Nuisance covariates included age and total intracranial volume.

| **Anatomical region** | **Cluster size (Voxels)** | **T value** | **Cluster-level P(FWE-corr)** | **x** | **y** | **z** |
| --- | --- | --- | --- | --- | --- | --- |
| Left Superior Temporal Gyrus | 10107 | 6.549 | 0 | -62 | -32 | 16 |
| Left Postcentral Gyrus |  |  |  | -62 | -18 | 20 |

**Supplementary Table 8.** **Demographic, clinical and musical background in stroke patients classified as fluent and dysfluent singers based on 1.5SD cut-off from mean fluency rate (words per minute) in spontaneous singing in the control group.** Mean (SD) data are presented unless otherwise indicated. Abbreviations: MBEA = Montreal Battery of Evaluation of Amusia. BDAE = Boston Diagnostic Aphasia Examination. WAB = Western Aphasia Battery. t= independent-samples t test. χ2 = chi-square test.

|  | **Fluent singers (N=23)** | **Dysfluent singers (N=20)** | **Difference between groups (P)** |
| --- | --- | --- | --- |
| **Demographic information** | | | |
| **Age** (years) | 65.6 (10.8) | 63.7 (9.6) | 0.547 (t) |
| **Gender** (female / male) | 16 / 7 | 9 / 11 | 0.103 (χ^2^) |
| **Handedness** (right/left/both) | 19 / 3 / 1 | 18 / 2 / 0 | 0.600 (χ^2^) |
| **Education** (years) | 13.3 (3.4) | 15.7 (4.6) | 0.057 (t) |
| **Musical background** | | | |
| **Choir singing** (years) | 4.6 (9.4) | 2.8 (10) | 0.554 (t) |
| **MBEA** | 22.7 (4.0) | 22.6 (4.7) | 0.913 (t) |
| **Clinical information** | | | |
| **Lesion size** (cm^3^) | 82.5 (81.2) | 132.4 (113.6) | 0.102 (t) |
| **Lesion laterality** (left/bilateral) | 17 / 6 | 17 / 3 | 0.373 (χ^2^) |
| **Time since stroke** (years) | 9.2 (8.6) | 8.7 (7.2) | 0.844 (t) |
| **BDAE severity score** | 3.5 (1.2) | 2.4 (1.4) | 0.006 (t) |
| **WAB indices** | | | |
| **Spontaneous speech** (max. score 20) | 15.7 (4.9) | 9.8 (7.9) | 0.006 (t) |
| **Repetition** (max. score 10) | 8.1 (2.6) | 4.4 (3.8) | < 0.001 (t) |
| **Naming** (max. score 10) | 7.7 (2.7) | 4.4 (3.8) | 0.002 (t) |
| **Spontaneous speech production (Correct Information Units per minute)** | | | |
| **Procedural question (Sunday) + Picture description (Picnic, Argument tasks)** | 33.3 (20.3) | 18.3 (19.9) | 0.019 (t) |
| **Singing production of Brother John (Correct Words per minute)** | | | |
| **Spontaneous singing** | 63.7 (18.1) | 17.0 (14.9) | < 0.001 (t) |
| **Speech repetition (Correct Words per minute)** | | | |
| **Spoken phrases** | 58.0 (18.9) | 29.7 (27.3) | < 0.001 (t) |
| **Sung phrases** | 30.9 (9.4) | 14.3 (13.9) | < 0.001 (t) |

**Supplementary Table 9.** **Grey matter volume clusters showing a significant positive correlation with words per minute in spontaneous singing of a familiar song in the group of dysfluent singers.** The x,y,z coordinates are according to the MNI atlas (mm). Nuisance covariates included age and total intracranial volume.

| **Anatomical region** | **Cluster size (Voxels)** | **T value** | **Cluster-level P(FWE-corr)** | **x** | **y** | **z** |
| --- | --- | --- | --- | --- | --- | --- |
| Left Middle Temporal Gyrus | 478 | 4.933 | 0.006 | -36 | -60 | 20 |

**Supplementary Table 10.** Lesion loadings in the 23 AAL Atlas regions for fluent (n=23) and dysfluent (n=20) singers.

| **AAL Atlas** | **Mean Fluent Singers** | **Mean Dysfluent Singers** | **P value (t)** | **P-adj (FDR < 0.05)** |
| --- | --- | --- | --- | --- |
| **Temporal_Sup_L*** | 0.132 | 0.427 | 0.002 | 0.023* |
| **Temporal_Pole_Sup_L*** | 0.037 | 0.305 | 0.001 | 0.023* |
| **Temporal_Mid_L*** | 0.065 | 0.296 | 0.006 | 0.046* |
| **Temporal_Pole_Mid_L** | 0.032 | 0.203 | 0.01 | 0.058 |
| **Frontal_Inf_Orb_L** | 0.074 | 0.279 | 0.016 | 0.074 |
| **Frontal_Inf_Tri_L** | 0.155 | 0.366 | 0.02 | 0.077 |
| **Rolandic_Oper_L** | 0.314 | 0.575 | 0.03 | 0.092 |
| **Insula_L** | 0.299 | 0.542 | 0.032 | 0.092 |
| **Heschl_L** | 0.252 | 0.499 | 0.04 | 0.102 |
| **Cingulum_Mid_L** | 0.092 | 0.007 | 0.047 | 0.105 |
| **Temporal_Inf_L** | 0.021 | 0.131 | 0.05 | 0.105 |
| **Frontal_Mid_Orb_L** | 0.04 | 0.173 | 0.059 | 0.108 |
| **Frontal_Inf_Oper_L** | 0.284 | 0.484 | 0.061 | 0.108 |
| **Frontal_Sup_Orb_L** | 0.018 | 0.102 | 0.111 | 0.182 |
| **Frontal_Med_Orb_L** | 0.006 | 0.066 | 0.181 | 0.278 |
| **Frontal_Mid_L** | 0.103 | 0.181 | 0.253 | 0.364 |
| **Cingulum_Post_L** | 0.021 | 0.001 | 0.33 | 0.446 |
| **Precentral_L** | 0.181 | 0.251 | 0.38 | 0.486 |
| **Postcentral_L** | 0.161 | 0.222 | 0.416 | 0.504 |
| **Supp_Motor_Area_L** | 0.089 | 0.04 | 0.449 | 0.516 |
| **Cingulum_Ant_L** | 0.035 | 0.019 | 0.517 | 0.566 |
| **Frontal_Sup_Medial_L** | 0.046 | 0.069 | 0.672 | 0.703 |
| **Frontal_Sup_L** | 0.076 | 0.081 | 0.937 | 0.937 |

**References**

1. H. Goodglass, Kaplan, E., *The Assessment of Aphasia and Related Disorders*. Waverly, Inc., Baltimore (Finnish version: Laine, M., Niemi, J., Koivuselkä-Sallinen, P., & Tuomainen, J. (1997) Afasian ja liitännäisoireiden arviointi, Psykologien kustannus Oy, Helsinki.), 1983.

2. L. E. Nicholas and R. H. Brookshire, "A system for quantifying the informativeness and efficiency of the connected speech of adults with aphasia," *J Speech Hear Res,* vol. 36, no. 2, pp. 338-50, Apr 1993, doi: 10.1044/jshr.3602.338.

3. A. Kertesz, *The Western aphasia battery*. New York: Grune & Stratton. (Finnish version (2005) WAB – Western aphasia battery. Psykologien kustannus Oy, Helsinki.) 1982.

4. A. Norton, L. Zipse, S. Marchina, and G. Schlaug, "Melodic intonation therapy: shared insights on how it is done and why it might help," *Ann N Y Acad Sci,* vol. 1169, pp. 431-6, Jul 2009, doi: 10.1111/j.1749-6632.2009.04859.x.

5. Y. Zhang, D. Y. Kimberg, H. B. Coslett, M. F. Schwartz, and Z. Wang, "Multivariate lesion-symptom mapping using support vector regression," *Hum Brain Mapp,* vol. 35, no. 12, pp. 5861-76, Dec 2014, doi: 10.1002/hbm.22590.

6. T. Straube, A. Schulz, K. Geipel, H. J. Mentzel, and W. H. Miltner, "Dissociation between singing and speaking in expressive aphasia: the role of song familiarity," *Neuropsychologia,* vol. 46, no. 5, pp. 1505-12, Apr 2008, doi: 10.1016/j.neuropsychologia.2008.01.008.

7. Y. H. Mah, M. Husain, G. Rees, and P. Nachev, "Human brain lesion-deficit inference remapped," *Brain,* vol. 137, no. Pt 9, pp. 2522-31, Sep 2014, doi: 10.1093/brain/awu164.

8. G. Hickok and D. Poeppel, "The cortical organization of speech processing," *Nat Rev Neurosci,* vol. 8, no. 5, pp. 393-402, May 2007, doi: 10.1038/nrn2113.

9. T. Särkämö, M. Tervaniemi, and M. Huotilainen, "Music perception and cognition: development, neural basis, and rehabilitative use of music," *Wiley Interdiscip Rev Cogn Sci,* vol. 4, no. 4, pp. 441-451, Jul 2013, doi: 10.1002/wcs.1237.

10. A. T. DeMarco and P. E. Turkeltaub, "A multivariate lesion symptom mapping toolbox and examination of lesion-volume biases and correction methods in lesion-symptom mapping," *Hum Brain Mapp,* vol. 39, no. 11, pp. 4169-4182, Nov 2018, doi: 10.1002/hbm.24289.

11. D. Y. Kimberg, H. B. Coslett, and M. F. Schwartz, "Power in Voxel-based lesion-symptom mapping," *J Cogn Neurosci,* vol. 19, no. 7, pp. 1067-80, Jul 2007, doi: 10.1162/jocn.2007.19.7.1067.

12. J. Ashburner and K. J. Friston, "Voxel-based morphometry--the methods," *Neuroimage,* vol. 11, no. 6 Pt 1, pp. 805-21, Jun 2000, doi: 10.1006/nimg.2000.0582.

13. J. Ashburner and K. J. Friston, "Unified segmentation," *Neuroimage,* vol. 26, no. 3, pp. 839-51, Jul 1 2005, doi: 10.1016/j.neuroimage.2005.02.018.

14. N. Tzourio-Mazoyer *et al.*, "Automated anatomical labeling of activations in SPM using a macroscopic anatomical parcellation of the MNI MRI single-subject brain," *Neuroimage,* vol. 15, no. 1, pp. 273-89, Jan 2002, doi: 10.1006/nimg.2001.0978.

15. Y. Benjamini and Y. Hochberg, "Controlling the False Discovery Rate: A Practical and Powerful Approach to Multiple Testing," *Journal of the Royal Statistical Society. Series B (Methodological),* vol. 57, no. 1, pp. 289-300, 1995.

16. L. Bonilha *et al.*, "Neural structures supporting spontaneous and assisted (entrained) speech fluency," *Brain,* vol. 142, no. 12, pp. 3951-3962, Dec 1 2019, doi: 10.1093/brain/awz309.

17. B. Kleber, Zarate, J.M., "The Neuroscience of Singing," in *The Oxford Handbook of Singing*, G. F. Welch, Howard, D.M., Nix, J. Ed. Oxford: Oxford University Press, 2019.

18. A. M. Zamorano, I. Cifre, P. Montoya, I. Riquelme, and B. Kleber, "Insula-based networks in professional musicians: Evidence for increased functional connectivity during resting state fMRI," *Human Brain Mapping,* vol. 38, no. 10, pp. 4834-4849, 2017/10/01 2017, doi: 10.1002/hbm.23682.
